# A Rapid Assessment Model for Liver Toxicity of Macrolides and an Integrative Evaluation for Azithromycin Impurities

**DOI:** 10.3389/fphar.2022.860702

**Published:** 2022-04-04

**Authors:** Miao-Qing Zhang, Jing-Pu Zhang, Chang-Qin Hu

**Affiliations:** ^1^ Key Laboratory of Biotechnology of Antibiotics, The National Health Commission (NHC), Beijing Key Laboratory of Antimicrobial Agents, Institute of Medicinal Biotechnology, Chinese Academy of Medical Sciences and Peking Union Medical College, Beijing, China; ^2^ National Institutes for Food and Drug Control, Beijing, China

**Keywords:** macrolides, impurity, liver toxicity, ADMET, docking energy score, prediction interval, *c-fos*, structure–hepatotoxicity relationship

## Abstract

Impurities in pharmaceuticals of potentially hazardous materials may cause drug safety problems. Macrolide antibiotic preparations include active pharmaceutical ingredients (APIs) and different types of impurities with similar structures, and the amount of these impurities is usually very low and difficult to be separated for toxicity evaluation. Our previous study indicated that hepatotoxicity induced by macrolides was correlated with c-fos overexpression. Here, we report an assessment of macrolide-related liver toxicity by ADMET prediction, molecular docking, structure–toxicity relationship, and experimental verification *via* detection of the *c-fos* gene expression in liver cells. The results showed that a rapid assessment model for the prediction of hepatotoxicity of macrolide antibiotics could be established by calculation of the -CDOCKER interaction energy score with the FosB/JunD bZIP domain and then confirmed by the detection of the *c-fos* gene expression in L02 cells. Telithromycin, a positive compound of liver toxicity, was used to verify the correctness of the model through comparative analysis of liver toxicity in zebrafish and cytotoxicity in L02 cells exposed to telithromycin and azithromycin. The prediction interval (48.1∼53.1) for quantitative hepatotoxicity in the model was calculated from the docking scores of seven macrolide antibiotics commonly used in clinics. We performed the prediction interval to virtual screening of azithromycin impurities with high hepatotoxicity and then experimentally confirmed by liver toxicity in zebrafish and *c-fos* gene expression. Simultaneously, we found the hepatotoxicity of azithromycin impurities may be related to the charge of nitrogen (N) atoms on the side chain group at the C5 position *via* structure–toxicity relationship of azithromycin impurities with different structures. This study provides a theoretical basis for improvement of the quality of macrolide antibiotics.

## Introduction

Macrolide antibiotics are commonly used for infectious diseases caused by bacterial pathogens (Farrington, 1998). In addition to antibacterial properties, macrolides have also displayed antiviral, antitumor, immunosuppressant, or other pharmacological effects (Labro, 2004; Prescott and Johnson, 2005; Asada et al., 2009; Spagnolo et al., 2013; Haworth et al., 2014). At present, macrolides have exhibited drug resistance along with widespread use in clinics, leading to many derivatives with better pharmacokinetic properties which have been synthesized (Chantot et al., 1986; Girard et al., 1987; Omura et al., 1992; Denis et al., 1999). However, an accompanying result shows that various impurities are produced in the chemical structure modifications, which may cause many drug safety problems as they are potentially hazardous materials. The impurities generally are in small amounts and cannot be easily separated, which makes the safety evaluation difficult, such as toxicity evaluation. Therefore, establishing a toxicity assessment paradigm of impurities is particularly important for drug quality management.

Traditionally, the toxicity assessment of a compound required at least thousands of laboratory animals. However, animal tests are tremendously expensive and time-consuming, which are the major limiting factors for evaluating chemical toxicity. Currently, it is recognized that a successful drug is determined not only by better efficacy but also by acceptable absorption, distribution, metabolism, excretion, and toxicity (ADMET) properties, leading to various *in vitro* high-throughput ADMET screening established in the early stages of drug discovery, which help reduce the cost of drug development and the number of safety problems (Merlot, 2010; Moroy et al., 2012). Structure–activity/toxicity relationship has been employed to investigate ADMET properties and predict toxicity *in silico* (Greene et al., 2015; Pavan et al., 2016; Guan et al., 2019). In addition, our recent studies have shown that structure–toxicity relationship combined with ADMET parameters evaluation and molecular docking (structure-based computations) can be used to predict drug impurity toxicity *in silico* (Han et al., 2017; Han et al., 2018a; Han et al., 2018b; Han et al., 2019).

Zebrafish has been commonly used as an animal model system to study toxicology. In recent years, zebrafish models have been widely used to evaluate liver toxicity (Zhang et al., 2017a; Huo et al., 2019). The structure, cellular composition, main physiological processes, function, and response to injury of zebrafish liver are found to be similar to those of the human liver (Hinton and Couch, 1998; Field et al., 2003; Goessling and Sadler, 2015). Zebrafish larvae are virtually transparent, enabling the development and the morphological changes in liver visible (Hill et al., 2012). Moreover, because of the rapid development and high fecundity, zebrafish models have been utilized for high-throughput screening (Vliegenthart et al., 2014). In our previous study, we established a zebrafish model of hepatotoxicity induced by macrolides and found that the hepatotoxicity induced by macrolides was related to the high expression of *c-fos* in the liver (Zhang et al., 2020). We speculated that the *c-fos* expression in the liver cells might be used to predict the hepatotoxicity of macrolides, while the liver injury could not be completely quantified using by this method. Therefore, a faster method to evaluate hepatotoxicity induced by macrolide compounds needs to be established.

Previous studies have shown that the *c-fos* level was elevated in acetaminophen (APAP)-induced liver injury, and the hepatocyte-specific deletion of *c-fos* showed hepatoprotective effects against APAP and diethylnitrosamine (DEN)-induced liver toxicity (Bakiri et al., 2017; Zhang et al., 2017b). *c-fos* is a member of the activator protein-1 (AP-1) transcription factor family, which harbors the basic leucine zipper domain (bZIP), and the bZIP gives AP-1 the ability to form the dimeric complex and bind DNA (Landschulz et al., 1988; Ellenberger et al., 1992). APAP-induced hepatotoxicity causes the high affinity of AP-1 on the DNA sequence, which results in increasing *c-fos* expression and AP-1 DNA binding activity (Blazka et al., 1996). Meanwhile, the main binding domain of AP-1 binding to DNA is FosB/JunD bZIP domain; thus, this domain is associated in the hepatotoxic response of the liver to APAP (Blazka et al., 1996; Yin et al., 2017). However, whether the liver toxicity induced by macrolides was associated with the FosB/JunD bZIP domain needs to be verified.

Macrolide antibiotic preparations not only include active pharmaceutical ingredients (APIs), but also include different types of impurities with similar structures are produced by APIs degradation or other interactions on the manufacture process or the storage. The amount of these impurities is usually very low and difficult to be separated for toxicity evaluation. In the present study, we established a rapid assessment model for quickly evaluating liver toxicity induced by macrolide compounds *via* prediction *in silico*, first by using molecular docking and then experimental verification by detecting the *c-fos* mRNA level in human liver cells and liver toxicity in zebrafish, which will be helpful for the drug safety assessment, especially for the impurities, quality control, and production process optimization of macrolide antibiotics.

## Materials and Methods

### Samples

The reference standards of macrolide antibiotics (erythromycin, roxithromycin, clarithromycin, azithromycin, impurity J, midecamycin, josamycin, and acetylspiramycin) and azithromycin impurities (impurities F, J, I, L, Q, R, and S) were obtained from the National Institutes for Food and Drug Control (NIFDC). The purity of all compounds was >95%.

### Visual Assessment of Liver Toxicity in Zebrafish

Wild-type AB strain zebrafish (*Danio rerio*) and transgenic Tg (fabp10: dsRed) zebrafish in this study were reared in a standard laboratory environment with a 14-h light/10-h dark cycle at 28 ± 1°C (Chen et al., 2019). As previously described (Zhang et al., 2020), 3 days post-fertilization (3 dpf), zebrafish larvae were exposed to compound solutions at different concentrations for 72 h. For visual assessment of liver toxicity in zebrafish, the tested zebrafish larvae were imaged by using a fluorescence microscope to observe the liver phenotype of zebrafish larvae, 20 larvae per group.

### ADMET Prediction

As previously described (Han et al., 2019; Zhang et al., 2020), the ADMET parameters were predicted by using the pKCSM online protocols (http://biosig.unimelb.edu.au/pkcsm/prediction). The topological polar surface area (TPSA) and the octanol–water partition coefficient (log*P*) were calculated by Molinspiration tools (http://www.molinspiration.com/. molinspiration.com/), the two chemical descriptors were primary factors of absorption. In this study, the absorption level depends on factors including water solubility and the apparent permeability coefficient indicated by colon cancer cell line (Caco-2). The distribution level depends on factors including the volume of distribution (VDss) and the blood–brain barrier (logBB). The metabolism of drugs was predicted based on two main isoforms of cytochrome P450 (CYP2D6 and CYP3A4 substrates). The excretion level indicated by total clearance was measured by the proportionality constant Cltot. The toxicity was predicted based on the hepatotoxicity. These pharmacokinetic parameters were calculated and examined for compliance with standard ranges based on human data.

### Molecular Docking

The docking procedure in the present investigation was performed by CDOCKER algorithm on Discovery Studio (DS) 2020 software. For ligand preparation, the 3D structures of macrolide compounds were extracted from the PubChem Compound database (https://www.ncbi.nlm.nih.gov/pccompound/). CHARMM force field was applied to minimize the energy of ligands structure. For protein preparation, the X-ray crystal structures of human FosB*/*JunD bZIP domain (PDB ID: 5VPB) were downloaded from Protein Data Bank (PDB) database (http://www.rcsb.org/pdb/home/home.do). Before docking, the structure of this domain was prepared by adding up the hydrogen atoms and removing water molecules. Ten molecular docking poses saved for each ligand were ranked according to -CDOCKER energy (Supplementary Tables S3, S4). The lowest energy structure was considered as the most stable conformation, thus, the optimum pose with the highest -CDOCKER interaction energy was selected to detect the interaction between macrolide compounds and FosB*/*JunD bZIP domain.

### RNA-Seq Analysis

Zebrafish larvae at three dpf were exposed to azithromycin, and its impurities in 2 mM and dissected liver of larvae were collected at six dpf for transcriptome sequencing. As previously described (Han et al., 2018a), the process of ribonucleic acid sequencing (RNA-seq) analysis was carried out by CapitalBio Corporation (Beijing, China). To identify the differentially expressed genes (DEGs) between the control and compound treatment group, the threshold of fold change ≥2 (upregulated gene) were established. Functional enrichment analysis was performed by Gene Ontology (GO) terms and Kyoto Encyclopedia of Genes and Genomes (KEGG) pathways under a significance threshold of *p*-value < 0.05. Cytoscape software was employed to map the drug–pathway network.

### Quantitative RT-PCR Analysis

As previously described by Zhang et al. (2020), the treated zebrafish larvae liver and human L02 cells were collected for the qRT-PCR analysis. Total RNA were extracted from L02 cells and the dissected liver of larvae by using TRIzol reagent (Sigma–Aldrich, Saint Louis, MO, United States) and synthesized cDNA using M-MLV reverse transcriptase (Promega, M1708). The reaction of qRT-PCR was performed by LightCycler480. The relative mRNA levels were calculated with *β-actin* as the reference gene using the comparative 2^ΔΔCT^ quantization method. The specific primers (5′-3′) for amplification in zebrafish are as follows: *fosab* (F: TTA​CCA​GCC​TTA​ACG​CCG​AC, R: TGG​ACC​ATC​CAC​TGC​AAG​TC), *β-actin* (F: CCG​TGA​CAT​CAA​GGA​GAA​G, R: ATA​CCG​CAA​GAT​TCC​ATA​CC). The primer sequences (5′-3′) for qRT-PCR in L02 cells are as follows: *c-fos* (F: TAC​TAC​CAC​TCA​CCC​GCA​GA, R: GGC​CTC​CTG​TCA​TGG​TCT​TC), *β-actin* (F: CAC​CAT​TGG​CAA​TGA​GCG​GTT​C, R: AGG​TCT​TTG​CGG​ATG​TCC​ACG​T).

### Cytotoxicity in Human L02 Cells

As previously described by Zhang et al. (2020), the human L02 cells were incubated with each macrolide compounds at different concentrations for 24 h. For cell viability, Cell Counting Kit-8 (CCK-8; Dojindo, CK04) was used by following the manufacturer’s protocol and the absorbance of each well was measured at 450 nm using a microplate reader. For cell toxicity, the level of lactate dehydrogenase (LDH) release was determined by LDH activity assay kits (Dojindo, CK12) according to the manufacturer’s specification, and the absorbance at 490 nm in each well was measured by a microplate reader.

### Data Treatment

All experiments were performed in triplicate. All the data were shown as mean ± SD, and statistical analyses were performed by GraphPad Prism software. The statistical comparisons were carried out by one-factor ANOVA, and *p*-values < 0.05 were considered significant.

## Results

### Analysis of Molecular Docking of Macrolides With FosB/JunD bZIP Domain

**FIGURE 1:**
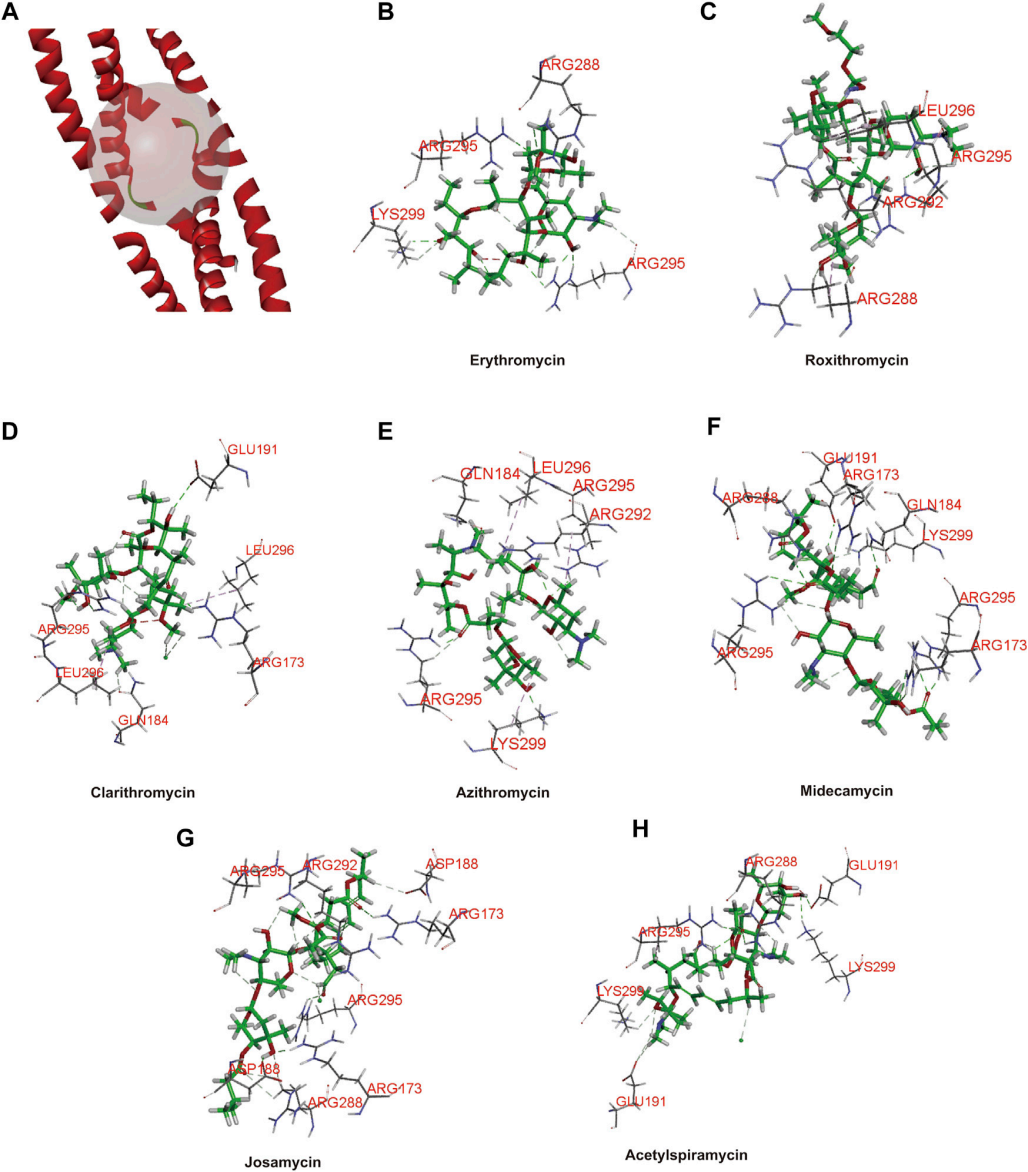
Three-dimensional (3D) structure model of protein ligand docking. **(A)** Active site of the FosB/JunD bZIP domain. **(B–H)** Docking interactions of erythromycin **(B)**, roxithromycin **(C)**, clarithromycin **(D)**, azithromycin **(E)**, midecamycin **(F)**, josamycin **(G)**, and acetylspiramycin **(H)** with the FosB/JunD bZIP domain. The representative images indicate the optimum docking poses in this domain.

Considering that *c-fos* is involved in some drug’s hepatotoxicity and its basic leucine zipper domain (bZIP) contributes to AP-1 binding to DNA (Zhang et al., 2020; Ellenberger et al., 1992; Landschulz et al., 1988), we infer that the interaction between the FosB/JunD bZIP domain and structures of drugs probably is a key access for understanding hepatotoxicity of macrolide drugs. Here, we used the FosB/JunD bZIP domain as *c-fos* receptor domain for macrolide molecular docking analysis. We selected erythromycin, roxithromycin, clarithromycin, azithromycin, midecamycin, josamycin, and acetylspiramycin as ligands to docking into the active site of the FosB/JunD bZIP domain. The most optimum docking poses in this domain are shown in Figure 1, and the highest -CDOCKER interaction energy scores are given in Table 1. The results showed that the scores of these seven macrolides were closely. -CDOCKER interaction energy score implies that the binding affinity between the ligand and the protein (Rampogu and Lemuel, 2016). The affinity order is azithromycin > erythromycin > acetylspiramycin > roxithromycin > midecamycin > clarithromycin > josamycin, which is in good agreement with the reports in literatures. The 16-membered ring macrolides were less hepatotoxic than erythromycin (Periti et al., 1993). Roxithromycin and clarithromycin have less hepatotoxicity than erythromycin, and azithromycin has higher hepatotoxicity than erythromycin (Björnsson, 2017). Our previous experimental data indicated that the liver toxicity induced by these seven compounds were similar (Zhang et al., 2020).

**TABLE 1:** Docking scores of the highest -CDOCKER interaction energy for the selected macrolide antibiotics.

Compound	-CDOCKER interaction energy (kcal/mol)
Erythromycin	51.10
Roxithromycin	50.11
Clarithromycin	49.98
Azithromycin	53.39
Midecamycin	50.07
Josamycin	49.24
Acetylspiramycin	50.20

**FIGURE 2:**
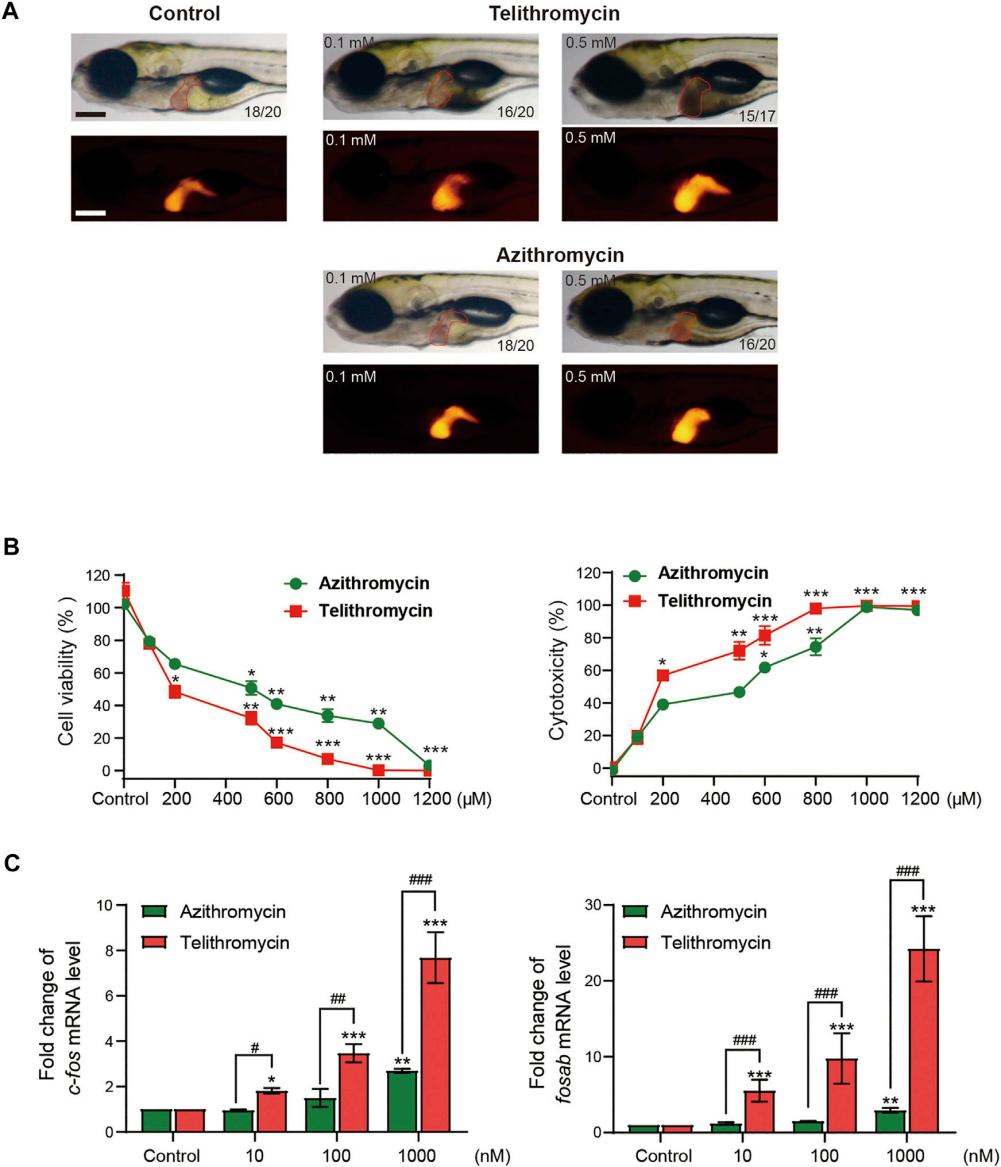
Toxicity of telithromycin and azithromycin in zebrafish liver and human liver cells. **(A)** Visual liver phenotype of zebrafish larvae at 6 dpf treated with telithromycin and azithromycin and imaged by a light microscopic (red outline) and a fluorescence microscopic (red fluorescence). The digit on the bottom right corner indicates the ratio of positive images. Scale bar, 250 μm. **(B)** Cytotoxicity of telithromycin and azithromycin on human L02 cells by using the CCK-8 kit to test cell viability and the LDH activity assay kit to detect cell cytotoxicity. **(C)** Expressions of the human *c-fos* gene were examined by qRT-PCR analysis in telithromycin and azithromycin treatment groups. Control, 0.1% DMSO-treated group, * indicates *p* < 0.05, ** indicates *p* < 0.01, *** indicates *p* < 0.001 compared to control group; # indicates *p* < 0.05, ## indicates *p* < 0.01, ### indicates *p* < 0.001 compared to the azithromycin group at a same concentration. All statistical data are given as mean ± SD (*n* = 3).

Then, we further confirmed the correlation between the docking energy scores and liver toxicity caused by macrolide antibiotics. It is well known that telithromycin is a ketolide antibiotic derived from macrolide structure (Supplementary Figure S1), has been pulled from the market due to severe acute liver injury (Author Anonymous, 2012). Therefore, we investigated the liver toxicity of telithromycin compared to azithromycin. First, employing transgenic zebrafish Tg (fabp10: dsRed), the liver phenotype *in vivo* showed that telithromycin could cause hepatomegaly at 0.1 mm and a dark coloration and amorphous liver at 0.5 mm; a dark and amorphous liver indicates degeneration or necrosis of liver cells. While azithromycin showed no toxicity to zebrafish liver at 0.1 mm, but hepatomegaly at 0.5 mm (Figure 2A). Thus, telithromycin has a higher liver injury than azithromycin in zebrafish. Second, the CCK-8 assay and LDH release assay were used to detect the cytotoxicity of telithromycin and azithromycin on human normal liver cells (L02 cells) (Figure 2B), the results suggested that telithromycin exerted a higher cytotoxicity than azithromycin in the human liver cells. Finally, the expression of *fosab* in zebrafish and *c-fos* in L02 cells were tested by using qRT-PCR analysis (Figure 2C), the results showed that telithromycin significantly elevated the mRNA levels of *fosab* and *c-fos* than azithromycin. These data demonstrated that the liver toxicity of telithromycin was higher than that of azithromycin.

Meanwhile, telithromycin as a ligand docked into the active site of FosB/JunD bZIP domain, and the docking score was 66.31 kcal/mol, which was much higher than that of azithromycin (Supplementary Figure S2; Supplementary Table S1). Therefore, the highest -CDOCKER interaction energy score with FosB/JunD bZIP domain may reflect the hepatotoxicity induced by macrolide antibiotics.

### A Prediction Interval for Rapid Evaluation of Hepatotoxicity Induced by Macrolides

To quickly assess the liver toxicity of diverse impurities of macrolides compared with their active pharmaceutical ingredients (APIs) in a time- or cost-saving manner, we formulated a prediction interval for hepatotoxicity according to the docking scores of macrolides binding with the FosB/JunD bZIP domain. The scope of the prediction interval was the mean ± 2 SD of docking scores of the selected seven representative macrolides (erythromycin, roxithromycin, clarithromycin, azithromycin, midecamycin, josamycin, and acetylspiramycin) commonly used clinically, which is 48.1∼53.1. When the score is less than 48.1, it is considered that the chemical has a little or no liver toxicity (less than API); when the score is in 48.1∼53.1, the liver toxicity is approximately equal to API; when the score is greater than 53.1, the liver toxicity is greater than API, and this impurity requires strict quality control.

Therefore, we might be able to put forward a hypothesis for the hepatotoxicity assessment model of macrolides: the virtual screening of impurities with high liver toxicity could be carried out quickly according to this prediction interval, and the effect on *c-fos* gene expression in L02 cells can be used as a complementary verification, and then the positive can be controlled as a specific impurity.

### Comparative Analysis of Hepatotoxicity of Azithromycin and Its Impurities by Using the Assessment Model

**FIGURE 3:**
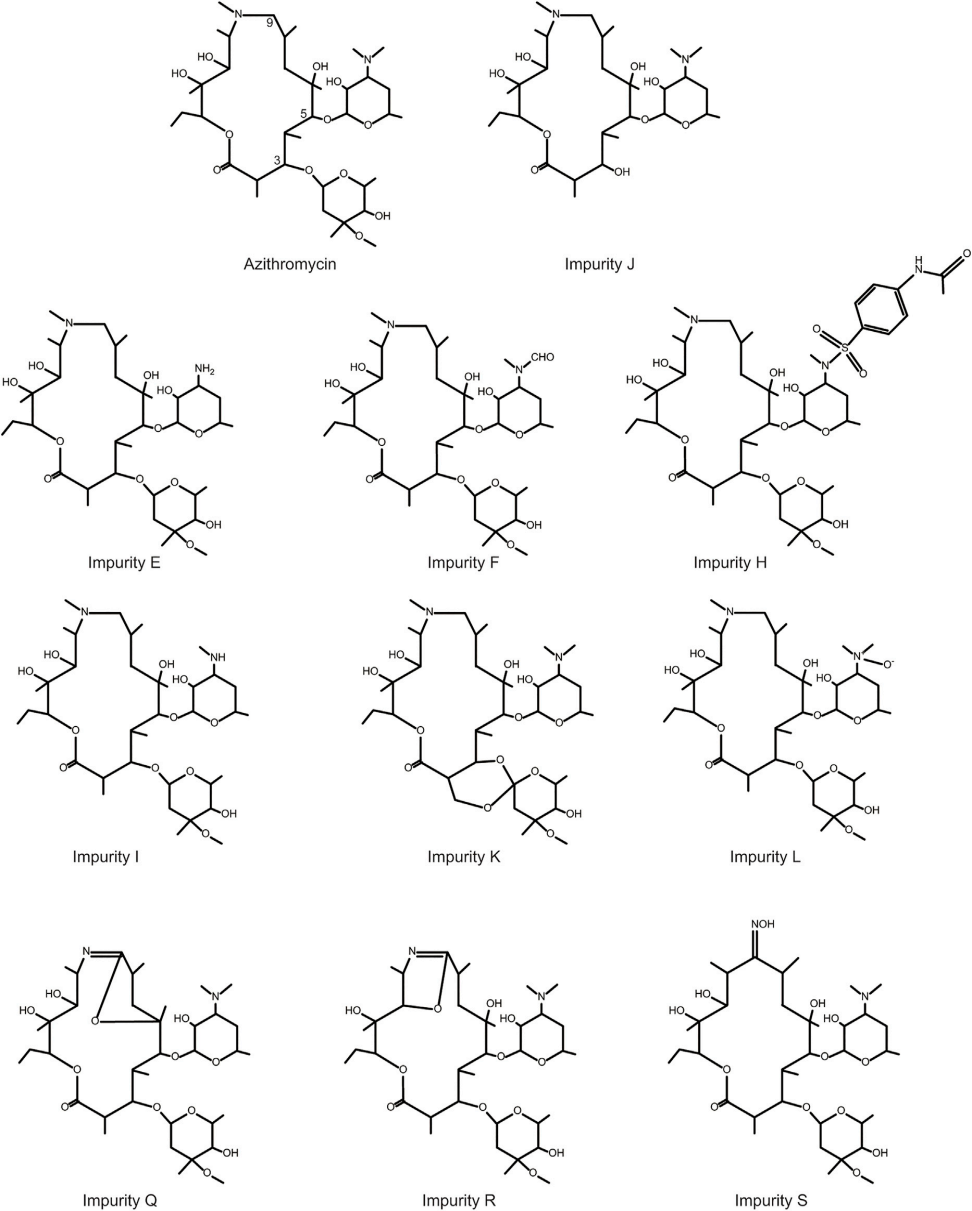
Chemical structures of azithromycin and its related impurities used in this study.

**FIGURE 4:**
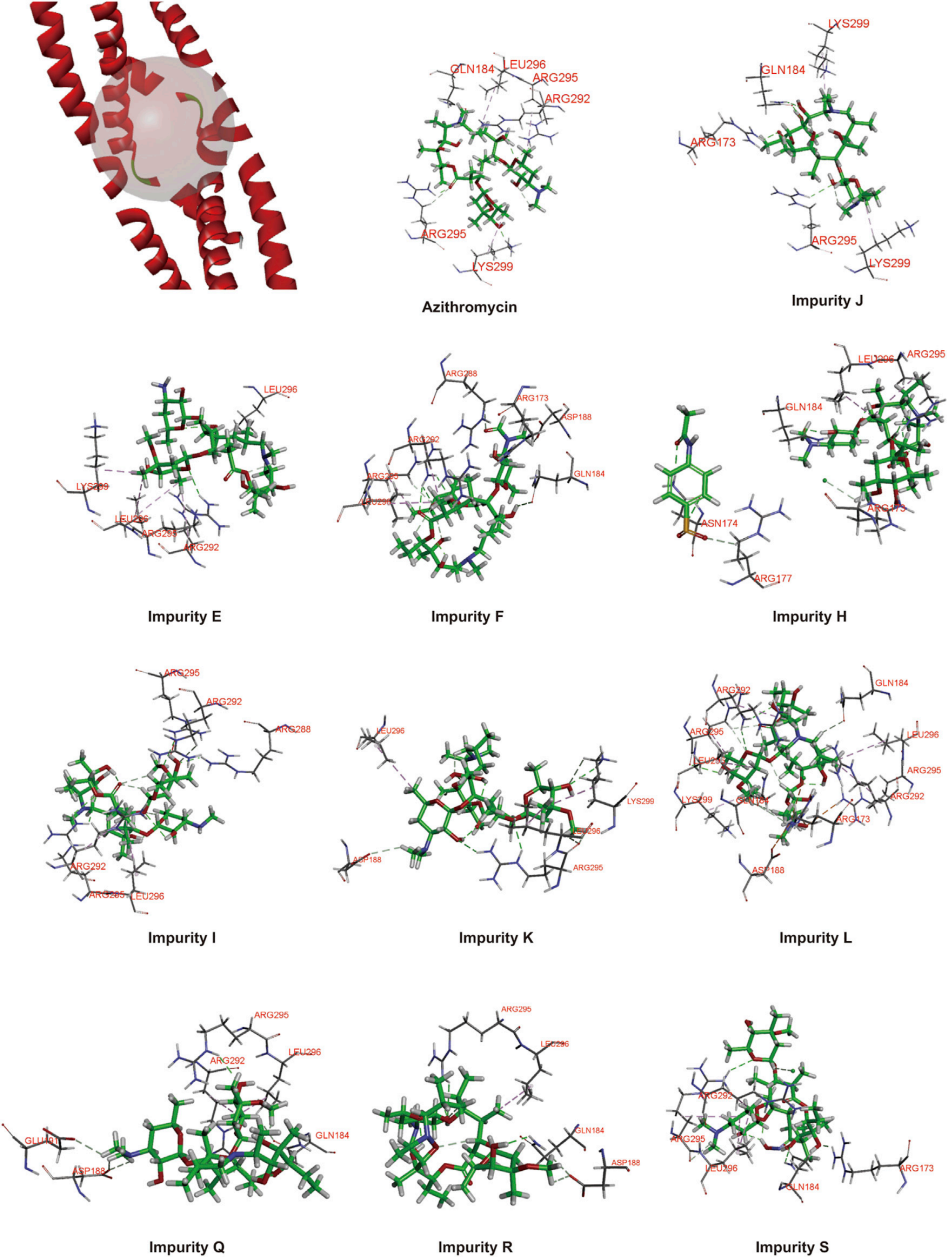
Protein ligand docking of azithromycin and its impurities with FosB/JunD bZIP domain.

In order to prove the aforementioned hypothesis and test the practicality of the hepatotoxicity assessment model of macrolides, first, we employed the prediction interval to virtually screen azithromycin impurities with high hepatotoxicity. We chose azithromycin and its impurities with different structures (impurities E, F, J, H, I, K, L, Q, R, and S) (Figure 3) as ligands to dock into the FosB/JunD bZIP domain. The most optimum docking poses of the azithromycin impurities are shown in Figure 4, and the highest -CDOCKER interaction energy scores are presented in Table 2. These scores revealed that the docking score of impurity H was the highest, followed by impurity L, both of which were greater than 53.1; the docking scores of impurities E, I, J, and Q were 48.1∼53.1; and the docking scores of impurities F, K, R, and S were less than 48.1. According to the prediction interval for hepatotoxicity of macrolides, we infer that impurities H and L might display higher hepatotoxicity than azithromycin; the hepatotoxicity of impurities E, I, J, and Q maybe equal to that of azithromycin, while the hepatotoxicity of impurities F, K, R, and S perhaps lower than that of azithromycin.

**TABLE 2:** Docking scores of the highest -CDOCKER interaction energy for azithromycin impurities.

Compound	-CDOCKER interaction energy (kcal/mol)
Impurity F	43.85
Impurity S	45.18
Impurity K	45.64
Impurity R	46.27
Impurity Q	48.81
Impurity E	50.13
Impurity I	51.74
Impurity J	50.42
Impurity L	55.57
Impurity H	66.45

**FIGURE 5:**
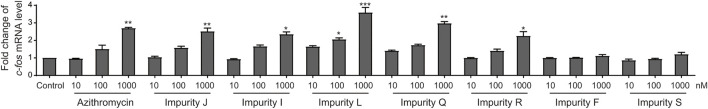
Azithromycin impurities J, I, L, Q, R, F, and S promote the expression human *c-fos* gene in L02 cells. The level of human *c-fos* mRNA were detected by qRT-PCR analysis in each treatment groups. Control, vehicle-treated group with 0.1% DMSO, * indicates *p* < 0.05, * indicates *p* < 0.01, *** indicates *p* < 0.001. All statistical data are represented as mean ± SD (*n* = 3).

Next, we detected the expression of *c-fos* gene in L02 cells to verify the results of virtual screening. The impurities F, J, I, L, Q, R, and S were successfully synthesized except for impurities E, H, and K, and were examined by qRT-PCR analysis. The results showed that impurities I, J, L, Q, and R significantly upregulated the expression of *c-fos* gene in L02 cells at a concentration dependent manner, and impurity L had the greatest effect, impurity R moderately lower than azithromycin, while F and S did not affect (Figure 5). These were consistent with the virtual screening results. Therefore, the hepatotoxicity prediction of azithromycin impurities may be accurate, implying feasibility of the hepatotoxicity assessment model for macrolides.

### An Integrative Evaluation of the Hepatotoxicity Induced by Azithromycin Impurities

To comprehensively analyze the hepatotoxicity induced by azithromycin impurities and further verify the results drawn by the assessment model, we investigated the liver toxicity of azithromycin impurities *in vivo* and *in vitro*. First, the ADMET properties of these impurities are predicted in Table 3, the values of topological polar surface area (TPSA) and log*P* showed that azithromycin impurities presented high lipophilicity, indicating these impurities might have good membrane permeability or oral absorption (Fonteh et al., 2015; Qidwai, 2016). Among them, impurity H has the highest TPSA and log *P* values. The absorption levels were assessed by water solubility and Caco2 permeability, the Caco2 permeability value of impurity L was the highest, and impurity H was the second. Therefore, impurity H and L had a better absorption than other impurities, which might lead to higher concentration *in vivo*. The volume of distribution at steady state (VDss) and blood–brain barrier membrane permeability (logBB) were used to assess the distribution level of these impurities, the VDss value of impurity K was the highest, and impurity H was the second. A high VDss value indicates that this compound is more inclined to accumulate in tissues. The logBB of all impurities was < −1, indicating these impurities did not easily cross the blood–brain barrier. The metabolism levels were evaluated by the two main subtypes of cytochrome P450, namely, CYP2D6 and CYP3A4. Impurities F, H, I, L, and Q were likely to be metabolized by CYP3A4, while all these impurities were not metabolized by CYP2D6. About the drug elimination level, the total clearance of impurity H was the lowest. For prediction of toxicity, all selected azithromycin impurities might cause hepatotoxicity.

**TABLE 3:** ADMET parameter prediction of azithromycin and its related impurities in this study.

Principal descriptor	Azithromycin	Impurity J	Impurity E	Impurity F	Impurity K	Impurity H	Impurity I	Impurity L	Impurity Q	Impurity R	Impurity S
TPSA (Å^2^)	180.09	152.39	202.88	197.16	189.32	243.33	188.88	193.92	178.22	178.22	209.44
Log *P* ^a^	1.9007	0.9755	1.2978	1.4273	1.2387	2.6182	1.5585	1.9133	2.4053	2.4053	2.0467
Absorption											
Water solubility (log mol/L)	−4.133	−3.918	−4.073	−4.069	−4.049	−3.246	−4.104	−4.331	−4.621	−4.588	−4.164
Caco2 permeability (log Papp in 10^−6^ cm/s)^b^	−0.211	−0.122	−0.228	0.154	−0.074	0.289	−0.227	0.29	−0.012	−0.055	−0.149
Distribution											
VDss (human, log L/kg)	−0.214	−0.09	−0.293	−0.43	0.245	0.065	−0.243	−0.514	−0.25	−0.151	−0.238
BBB permeability (logBB)	−1.857	−1.263	−1.772	−1.959	−1.847	−2.409	−1.856	−2.018	−1.677	−1.681	−1.882
Metabolism											
CYP2D6 substrate	No	No	No	No	No	No	No	No	No	No	No
CYP3A4 substrate	Yes	No	No	Yes	No	Yes	Yes	Yes	Yes	No	No
Excretion											
Total clearance [log (ml/min/kg)]^c^	−0.424	−0.472	−0.349	−0.271	−0.53	−0.824	−0.292	−0.426	−0.219	−0.133	−0.661
Toxicity											
Hepatotoxicity	Yes	Yes	Yes	Yes	Yes	Yes	Yes	Yes	Yes	Yes	Yes

a Octanol/water partition coefficient.

b Logarithm of the apparent permeability coefficient.

c Drug clearance is measured by the proportionality constant CLtot.

**FIGURE 6:**
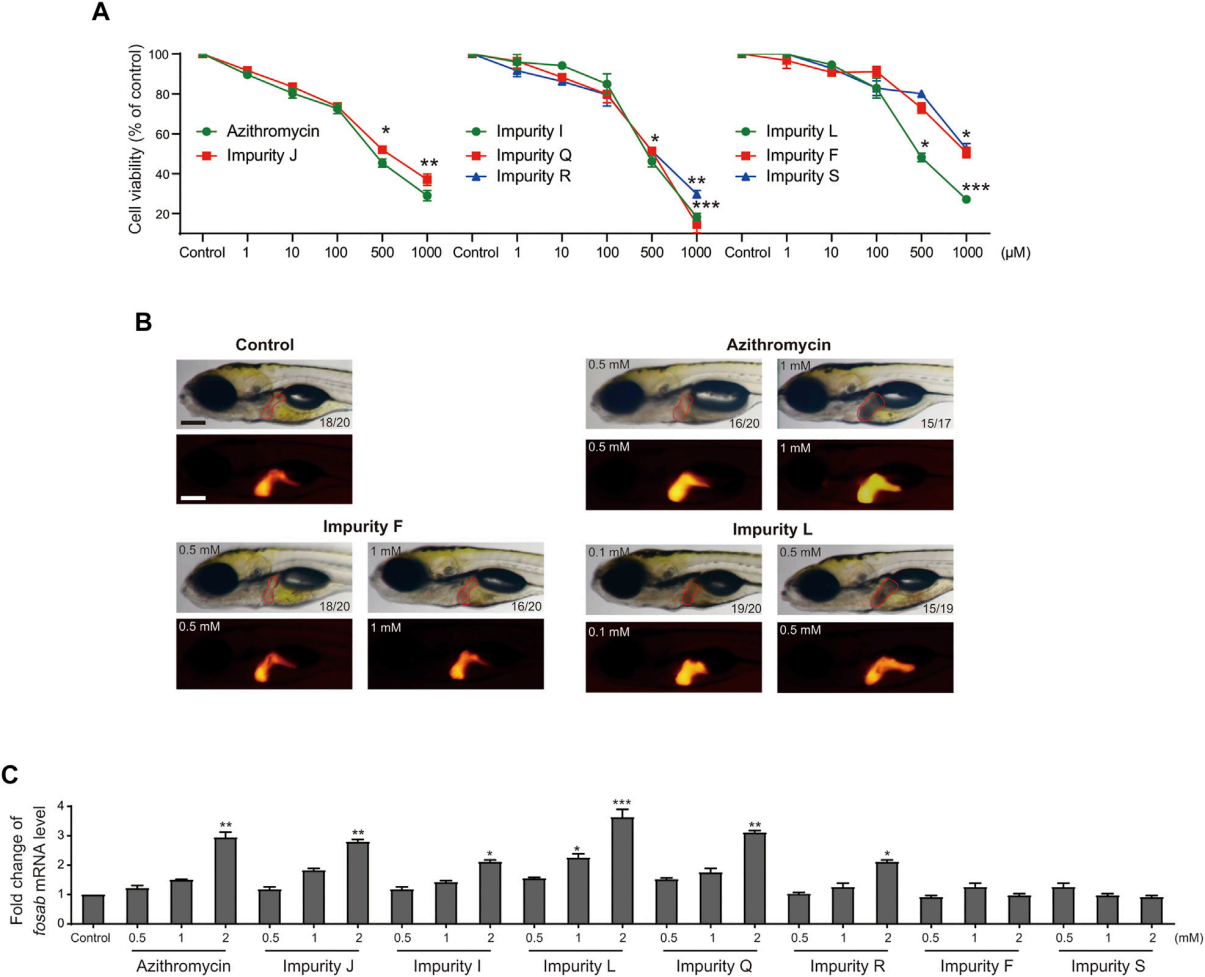
Analysis of the liver toxicity of azithromycin impurities *in vitro* and *in vivo*. **(A)** Cell viability of L02 cells after exposed to azithromycin and impurities J, I, L, Q, R, F, and S solutions were tested by CCK-8 assay. Control indicates 0.1% DMSO-treated group. * indicates *p* < 0.05, ** indicates *p* < 0.01, ***indicates *p* < 0.001 compared with the control group. All values are expressed as the mean ± SD (*n* = 3). **(B)** Visual phenotype of liver toxicity in zebrafish larvae at 6 dpf after azithromycin, impurities F and L treatment. Representative images of treated larvae by using a light microscopic (red outline) and a fluorescence microscopic (red fluorescence). The digit on the bottom right corner shows the ratio of positive images. Scale bar, 250 μm. **(C)** *fosab* mRNA levels in zebrafish exposed to azithromycin and its seven impurities, respectively, were detected by qRT-PCR analysis. Control, vehicle-treated group with 0.1% DMSO, * indicates *p* < 0.05, * indicates *p* < 0.01, *** indicates *p* < 0.001. All statistical data are represented as mean ± SD (*n* = 3).

Second, we detected the cytotoxicity of these impurities in L02 cells *in vitro via* CCK-8 assay to test the cell viability (Figure 6A). The results showed that all the impurities and azithromycin reduced the cell viability in a dose dependent manner. There were no significant differences in cytotoxicity of these impurities compared with azithromycin, except for impurity F and impurity S displaying less cytotoxicity in L02 cells, respectively. Furthermore, we chose impurity L, a potentially higher hepatotoxicity impurity, and impurity F, a possibly lower hepatotoxicity impurity, to assess the liver toxicity compared with azithromycin using the transgenic zebrafish (Figure 6B). The results demonstrated that impurity F was nearly non-toxic to zebrafish liver at 0.5 and 1 mm; azithromycin initiated hepatomegaly at 0.5 mm and blackened liver tissue at 1 mm; meanwhile, impurity L triggered hepatomegaly and caused the liver lost transparency with a brown coloration at 0.1 mm, and the liver tissue became black at 0.5 mm. These results demonstrated that impurity F and impurity S had a lower liver toxicity than azithromycin, whereas the liver toxicity of impurity L was higher than azithromycin. The results of liver toxicity *in vitro* and *in vivo* were consistent with the results evaluated by the assessment model. Importantly, these experimental data also provided proof for the accuracy and practicality of the assessment model for hepatotoxicity induced by macrolides.

Furthermore, RNA-seq and qRT-PCR analyses were performed to examine the expression of *c-fos* gene *in vivo*. According to GO enrichment analysis, we found *fosab* mRNA level were upregulated significantly by azithromycin impurities J, I, L, Q, and R, respectively, while impurity F and S had no significant effect on the expression of *fosab* in zebrafish (Table 4). The fold change of *fosab* gene expression promoted by impurity L was the highest. The results of qRT-PCR analysis also demonstrated that these impurities could significantly elevate the expression of *fosab* gene in zebrafish liver at a concentration dependent manner except for impurities F and S, while impurity L has the greatest effect (Figure 6C). These data were similar to the expression of *c-fos* gene in L02 cells. On the other hand, our previous study has shown that the liver toxicity induced by macrolides was related to the high expression of *c-fos* (Zhang et al., 2020), these data might explain why impurity L has a higher liver toxicity, probably due to its greater effect on *c-fos* gene expression. Therefore, the assessment model is accurate, which can be illustrated by the experimental confirmation via detection of the expression of *c-fos* gene in L02 cells.

**TABLE 4:** Fold-change of zebrafish *fosab* gene upregulated by azithromycin and its impurities J, I, Q, R, and L in RNA-seq analysis.

Gene	Fold change					
	Azithromycin	Impurity J	Impurity I	Impurity Q	Impurity R	Impurity L
*fosab*	3.27	2.92	2.14	3.07	2.18	4.10

### Structure–Hepatotoxicity Relationship of Azithromycin Impurities

**FIGURE 7:**
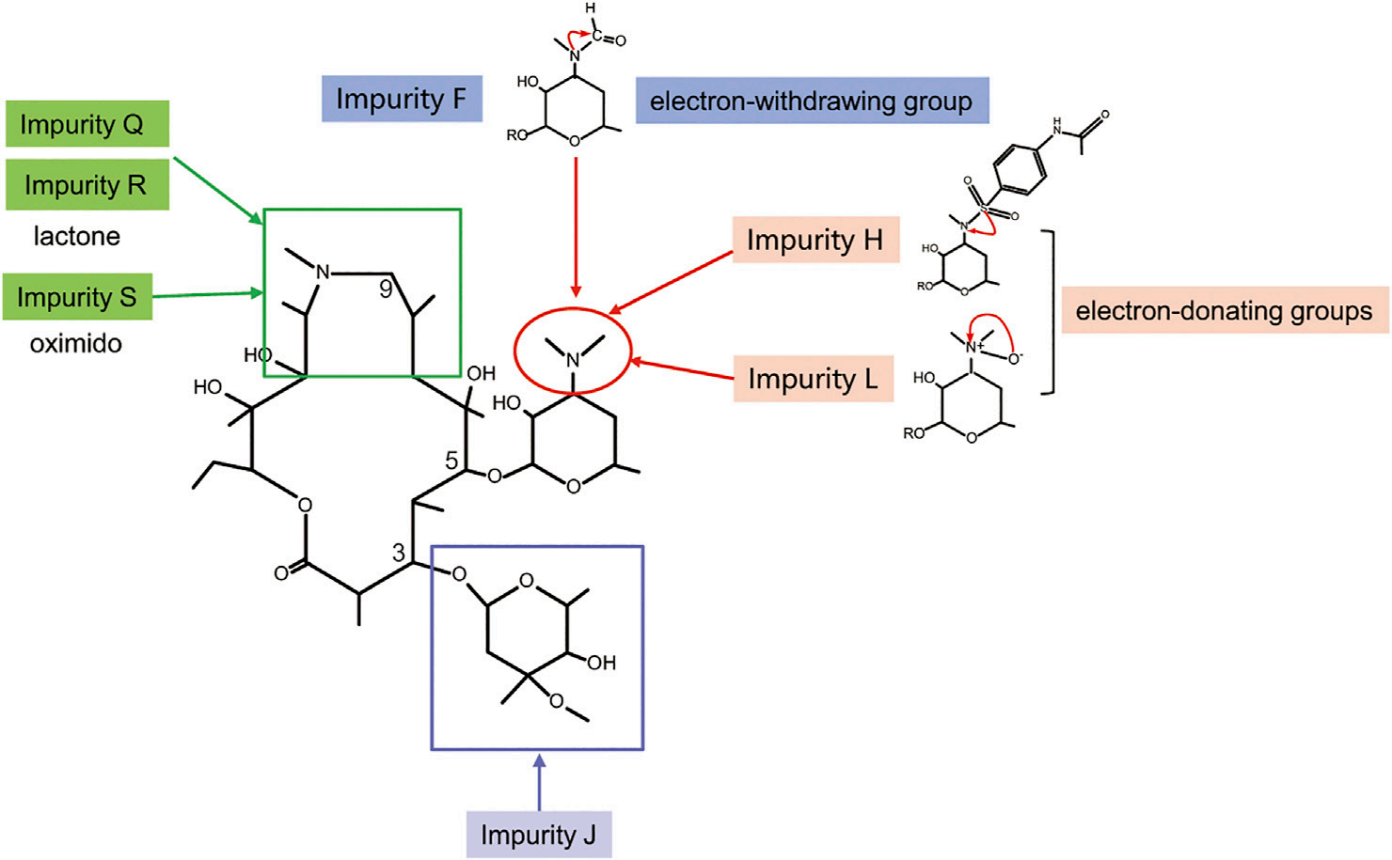
Structure–hepatotoxicity relationship of azithromycin impurities.

Furthermore, we performed comparative analyses of the structures of azithromycin impurities on the basis of the structural characteristics (Figure 3). The structures of these impurities have mainly changed in the side chain at C3, C5, and C9 position of lactone rings. Among them, the structures of impurities F, L, and H are different only in the nitrogen (N)-linked substituents on the side chain group at C5 position compared with azithromycin. The N-linked substituents in impurity F is an aldehyde group, which is an electron-withdrawing group from N atom, while the N-connected substituents in impurity H and impurity L are electron-donating groups, which lead to different charges of N atoms (Figure 7). Consequently, the hepatotoxicity of azithromycin impurities may be related to the charge of N atoms on the side chain group at C5 position through the analysis of the structure–hepatotoxicity relationship of azithromycin impurities. Simultaneously, the structural modification of C9 position to form lactone or oximido cannot exacerbate the hepatotoxicity according to the structures of impurities Q, R, and S, and the oximido of impurity S dramatically reduces hepatotoxicity. Similarly, the changes at C3 side chain also cannot aggravate the hepatotoxicity according to the structures of impurities K and J.

All the aforementioned results indicate that the assessment model for evaluating hepatotoxicity of macrolides may be accurate and practical, besides, the charge of N atoms on the side chain group at C5 position could also reflect the hepatotoxicity of macrolides.

## Discussion

Macrolide antibiotics products in the market have various sorts of impurities owing to different preparation or production process, while the structures of impurities are usually similar to API, the content of impurities are very low which makes it difficult to be separated for animal experiments. Whether these impurities play roles in the hepatotoxicity caused by administration of macrolides, which impurities should be controlled strictly or leniently, these questions are particularly important for the consistency evaluation between domestic generic drugs and brand-name drugs. On the basis of the structures of impurities, *in silico* prediction for toxicity by using structure–toxicity relationship, *in silico* ADMET prediction and molecular docking techniques has been widely used (Han et al., 2017; Han et al., 2018a; Han et al., 2018b; Han et al., 2019; Liu et al., 2019).

In this study, we studied whether the binding ability with FosB*/*JunD bZIP domain could quantify the hepatotoxicity of macrolides using telithromycin with severe liver toxicity and azithromycin. The docking value of telithromycin into FosB*/*JunD bZIP domain was much higher than azithromycin. Then, we used the following assays to examine the result. Two experimental tests showed that the hepatotoxicity of telithromycin was higher than that of azithromycin in zebrafish liver and cytotoxicity in human L02 cells. ADMET prediction showed that the absorption and distribution levels of telithromycin were also much higher than azithromycin, which might be one of the reasons for the high hepatotoxicity of telithromycin (Supplementary Table S2). Hence, the highest -CDOCKER interaction energy scores can indeed reflect the hepatotoxicity induced by macrolides, which implying the interaction between the FosB*/*JunD bZIP domain and macrolides probably involved in the liver toxicity of macrolides. Then, we established a prediction interval for hepatotoxicity according to the mean ± 2SD of the highest–CDOCKER interaction energy scores of representative macrolides, namely 48.1∼53.1.

Our previous study has shown the liver toxicity induced by macrolides related to high expression of *c-fos* (Zhang et al., 2020), we established a rapid assessment model for hepatotoxicity evaluation of macrolides *via* the prediction interval and detecting the expression of *c-fos* gene in human liver cells. In order to explore the practicability of the assessment model, we compared and analyzed the hepatotoxicity of azithromycin impurities. The predicted results by using the prediction interval were consistent with all the experimental results, indicating that the assessment model for evaluating hepatotoxicity of macrolides may be accurate and practical.

In addition, for a more comprehensive analysis, the results of kyoto encyclopedia of genes and genomes (KEGG) pathway enrichment analysis suggested these synthesized impurities and azithromycin co-regulated only in two significant pathways involving upregulated *fosab* gene, namely, apoptosis signaling pathway and insulin/IGF pathway-mitogen activated protein kinase/MAP kinase cascade pathway (Supplementary Figure S3), which was common with our previous results (Zhang et al., 2020). *fosab* encoded proto-oncogene *c-fos*. Therefore, the liver toxicity induced by azithromycin impurities might be also related to the high expression of *c-fos* gene. Moreover, this study also provides warning that impurities H and L might have serious hepatotoxicity and need to be strictly controlled.

Before evaluating the safety of a drug in clinic, ADMET assessment is usually performed for preliminary prediction. The liver toxicity of macrolides *in vivo* was determined by zebrafish hepatotoxicity model in this study, while ADME profiles are not easy to quantify in zebrafish. Recent studies confirmed that the absorption, distribution, and metabolism of drugs in zebrafish were consistent with those in mammals (Fischer et al., 2013; Fleming et al., 2013; Poon et al., 2017; Liu et al., 2019). Meanwhile, we used *in silico* ADME profile to predict and contrast the ADME parameters of azithromycin impurities in the present study. These data indicated that impurities H and L had a higher absorption. Our previous studies have shown that the toxic effects of compounds were closely related to the toxic functional groups and absorption of compounds (Zhang et al., 2013; Zhang et al., 2015; Chen et al., 2017). Thus, the results of ADME prediction give an explanation for the larger hepatotoxicity of impurities H and L, which may have resulted from their high absorption *in vivo*.

More importantly, we analyzed the structure–hepatotoxicity relationship of azithromycin impurities and found that the charge of N atoms on the side chain group at the C5 position might also affect the liver toxicity of these impurities. Macrolides are composed of a macrocytic lactone with different ring sizes; thus, the structure–hepatotoxicity relationship of azithromycin impurities could also be applicable to the other impurities of 14-membered ring and 16-membered ring macrolides (erythromycin, roxithromycin, clarithromycin, midecamycin, josamycin, and acetylspiramycin). Therefore, we can design a protocol to evaluate the hepatotoxicity of macrolide impurities (Supplementary Figure S4): first, observing the charge of N atoms on the side chain group at the C5 position; second, docking into the FosB*/*JunD bZIP domain to preliminary screening of impurities with greater hepatotoxicity than API by the prediction interval; and finally, detecting the expression of the *c-fos* gene in human L02 cells, and the positive impurity should be controlled as a specific impurity needed to be controlled.

In summary, we established a rapid assessment model to evaluate the hepatotoxicity of extensive impurities of 14-, 15-, and 16-membered ring macrolide antibiotics, which will provide a theoretical basis for quality consistency evaluation, manufacturing process improvement of macrolide antibiotics.

## Data Availability

The datasets presented in this study can be found in online repositories. The names of the repository/repositories and accession number(s) can be found at: https://www.ncbi.nlm.nih.gov/geo/; GSE195903.
