# Supplementary material for: A Rapid Assessment Model for Liver Toxicity of Macrolides and an Integrative Evaluation for Azithromycin Impurities
Source: Front Pharmacol. 2022 Apr 4;13:860702. doi: 10.3389/fphar.2022.860702 (PMC9014295; doi:10.3389/fphar.2022.860702)
Supplement: Supplementary file 1 [file DataSheet1.PDF]

Supplementary materials

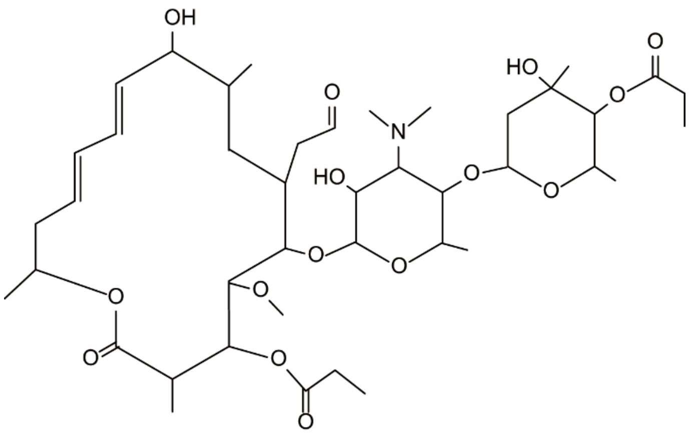

Figure S1. Chemical structure of telithromycin.

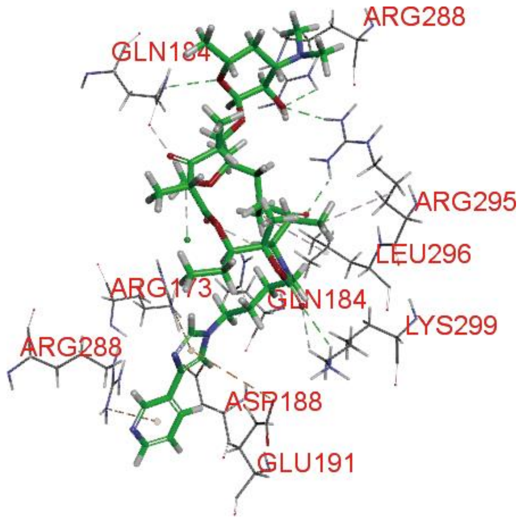

Figure S2. Protein ligand docking of telithromycin with FosB/JunD bZIP domain.

Table S1. The docking scores of the highest -CDOCKER interaction energy for telithromycin and azithromycin.

**Compounds**	**-CDOCKER Interaction Energy**
Azithromycin	53.39
Telithromycin	66.31

Table S2. ADMET parameters prediction of azithromycin and telithromycin *in silico*.

Principal descriptor	Azithromycin	Talithromycin
TPSA (Å2)	180.09	171.88
log*P*	1.9007	4.9292
**Absorption**		
Water solubility (log mol/L)	-4.133	-2.989
Caco2 permeability (log Papp in 10^{-6} cm/s)	-0.211	0.178
**Distribution**		
VDss (human, log L/kg)	-0.214	0.878
BBB permeability (logBB)	-1.857	-2.309
**Metabolism**		
CYP2D6 substrate	No	No
CYP3A4 substrate	Yes	Yes
**Excretion**		
Total Clearance (log(ml/min/kg))	-0.424	-0.224
**Toxicity**		
Hepatotoxicity	Yes	Yes

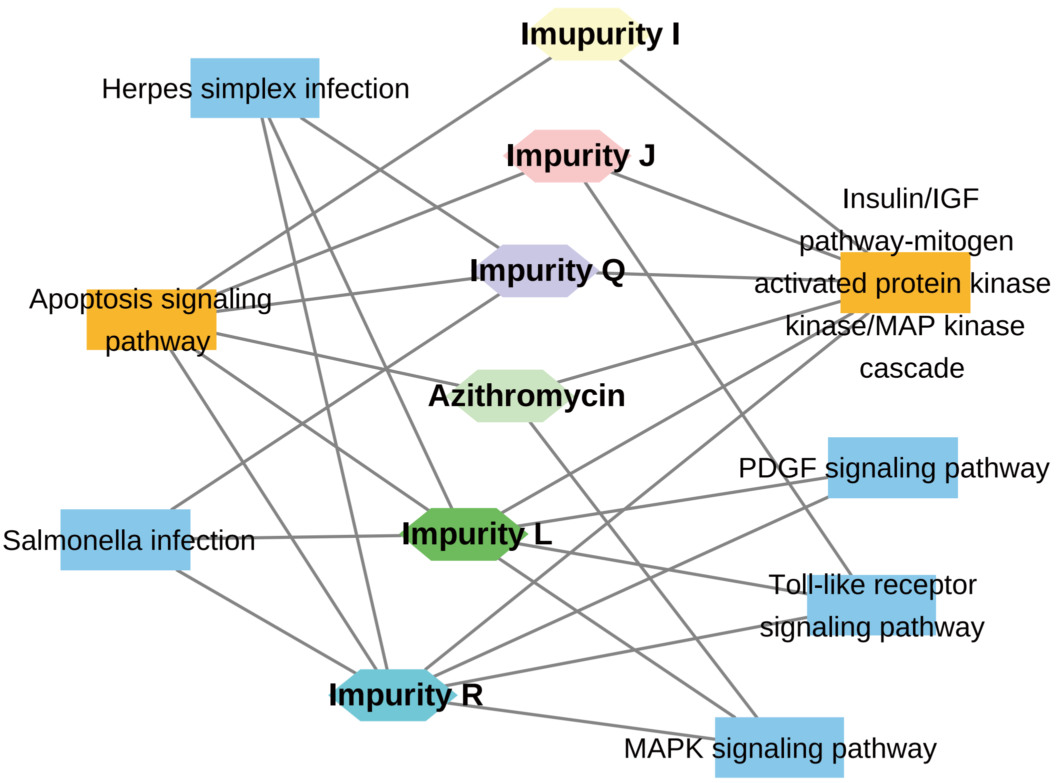

Figure S3. The “drug-pathway” network associated with *fosab* gene of azithromycin, impurity J, I, Q, R and L. Significantly enriched pathways involving *fosab* gene up-regulated in azithromycin and its synthesized impurities treatment groups were determined under a significance threshold of $P < 0.05$.

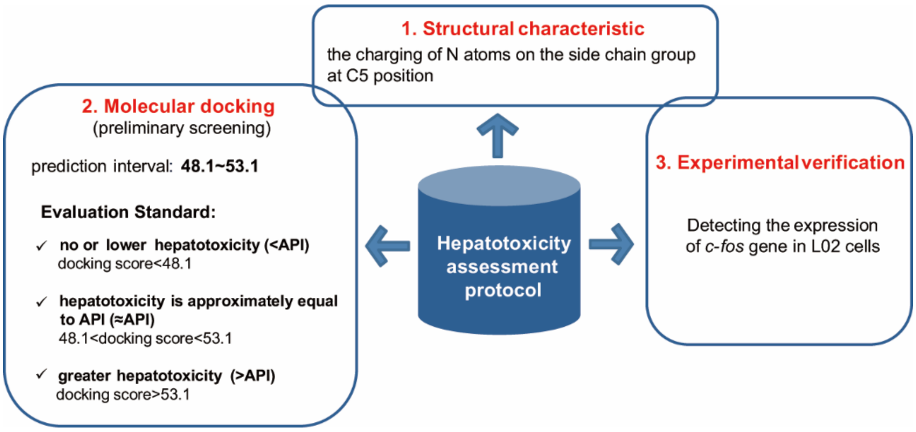

Figure S4. A protocol to evaluate the hepatotoxicity of macrolides.

Table S3. The docking scores of ten molecular docking poses for each macrolide antibiotics

Compounds	-CDOCKER Interaction Energy	Compounds	-CDOCKER Interaction Energy
Erythromycin	51.10	Midecamycin	50.07
Erythromycin	49.98	Midecamycin	48.48
Erythromycin	48.71	Midecamycin	47.93
Erythromycin	47.42	Midecamycin	47.50
Erythromycin	46.10	Midecamycin	44.36
Erythromycin	44.19	Midecamycin	42.80
Erythromycin	43.65	Midecamycin	42.00
Erythromycin	43.20	Midecamycin	40.30
Erythromycin	42.95	Midecamycin	33.13
Erythromycin	38.84	Midecamycin	33.04
Roxithromycin	50.11	Josamycin	49.24
Roxithromycin	48.79	Josamycin	47.19
Roxithromycin	46.73	Josamycin	44.62
Roxithromycin	42.30	Josamycin	44.34
Roxithromycin	42.07	Josamycin	41.69
Roxithromycin	40.74	Josamycin	41.65
Roxithromycin	40.24	Josamycin	39.88
Roxithromycin	40.21	Josamycin	38.00
Roxithromycin	36.88	Josamycin	36.68
Roxithromycin	34.53	Josamycin	33.26
Clarithromycin	49.98	Acetylspiramycin	50.20
Clarithromycin	47.49	Acetylspiramycin	47.88
Clarithromycin	47.46	Acetylspiramycin	46.80
Clarithromycin	46.93	Acetylspiramycin	46.41
Clarithromycin	44.13	Acetylspiramycin	46.15
Clarithromycin	42.64	Acetylspiramycin	45.71
Clarithromycin	40.42	Acetylspiramycin	42.12
Clarithromycin	40.08	Acetylspiramycin	40.88
Clarithromycin	39.01	Acetylspiramycin	36.65
Clarithromycin	38.14	Acetylspiramycin	33.96
Azithromycin	53.39	Telithromycin	66.31
Azithromycin	51.95	Telithromycin	63.91
Azithromycin	51.06	Telithromycin	63.19
Azithromycin	50.69	Telithromycin	62.87
Azithromycin	50.44	Telithromycin	59.10
Azithromycin	50.00	Telithromycin	59.06
Azithromycin	46.84	Telithromycin	57.90
Azithromycin	45.91	Telithromycin	57.88
Azithromycin	45.46	Telithromycin	55.17
Azithromycin	44.17	Telithromycin	52.18

Table S4. The docking scores of ten molecular docking poses for each azithromycin impurities.

Compounds	-CDOCKER Interaction Energy	Compounds	-CDOCKER Interaction Energy
Impurity F	43.85	Impurity E	50.13
Impurity F	40.99	Impurity E	50.10
Impurity F	40.78	Impurity E	47.63
Impurity F	40.40	Impurity E	47.52
Impurity F	38.30	Impurity E	45.66
Impurity F	37.66	Impurity E	44.59
Impurity F	37.36	Impurity E	43.25
Impurity F	37.35	Impurity E	42.70
Impurity F	36.56	Impurity E	37.81
Impurity F	30.86	Impurity E	37.56
Impurity S	45.18	Impurity I	51.74
Impurity S	43.24	Impurity I	49.64
Impurity S	42.99	Impurity I	46.04
Impurity S	40.64	Impurity I	45.75
Impurity S	40.63	Impurity I	42.62
Impurity S	40.17	Impurity I	40.21
Impurity S	39.77	Impurity I	40.16
Impurity S	39.57	Impurity I	38.19
Impurity S	39.45	Impurity I	35.75
Impurity S	39.42	Impurity I	31.59
Impurity K	45.64	Impurity J	50.42
Impurity K	44.93	Impurity J	50.19
Impurity K	44.92	Impurity J	50.13
Impurity K	44.76	Impurity J	49.58
Impurity K	41.62	Impurity J	46.31
Impurity K	41.30	Impurity J	45.67
Impurity K	40.02	Impurity J	45.65
Impurity K	38.29	Impurity J	44.64
Impurity K	37.06	Impurity J	43.80
Impurity K	36.08	Impurity J	40.60
Impurity R	46.27	Impurity L	55.57
Impurity R	45.94	Impurity L	55.33
Impurity R	45.83	Impurity L	55.01
Impurity R	45.65	Impurity L	50.40
Impurity R	45.45	Impurity L	49.47
Impurity R	43.55	Impurity L	47.57
Impurity R	42.25	Impurity L	47.24
Impurity R	40.86	Impurity L	37.87
Impurity R	40.71	Impurity L	35.83

Impurity R	38.06	Impurity L	35.53
Impurity Q	48.81	Impurity H	66.45
Impurity Q	48.11	Impurity H	66.16
Impurity Q	47.45	Impurity H	65.44
Impurity Q	45.69	Impurity H	64.70
Impurity Q	45.22	Impurity H	62.23
Impurity Q	42.61	Impurity H	58.19
Impurity Q	42.09	Impurity H	57.96
Impurity Q	41.25	Impurity H	57.75
Impurity Q	40.29	Impurity H	56.99
Impurity Q	34.51	Impurity H	56.93
